# Liberal Intravenous Fluid Administration in a Rare Case of Severe Rhabdomyolysis Secondary to SARS-CoV-2

**DOI:** 10.7759/cureus.17234

**Published:** 2021-08-16

**Authors:** Celine Fadel, Ngoc Phan, Aman Kaur

**Affiliations:** 1 Internal Medicine, Northeast Georgia Medical Center Gainsville, Gainesville, USA

**Keywords:** hepatocellular injury, rare disease, rhabdomyolysis, covid-19, sars-2-coronavirus

## Abstract

SARS-CoV-2 entered the world by storm when it made its appearance at the end of 2019 in Wuhan, China. The severity can range from asymptomatic infection, which occurs in approximately 33% of infected patients, to death. Worldwide deaths due to SARS-CoV-2 are currently approximated at 3.8 million people with close to 600,000 deaths in the United States alone, reiterating the significant impact this virus has on the population. SARS-CoV-2 can affect systems of the body such as respiratory, gastrointestinal tract, neurological, cardiac, renal, and even skeletal muscle tissue. A few cases of rhabdomyolysis are reported in SARS-CoV-2 infection, but the significant level of creatinine kinase in the hundreds of thousands is rare. Our case demonstrates the rarity of SARS-CoV-2 manifestation in a 33-year-old African American male with severe rhabdomyolysis with a creatinine kinase on the admission of 362,445 IU/L. The patient was treated aggressively with intravenous fluids, monitoring electrolytes, renal function, and respiratory status closely. His management includes liberal administration of fluid to treat his rhabdomyolysis, without compromising his respiratory status. He was subsequently discharged home after seven days of hospitalization. We strive to share this information in hopes to share our management for future similar cases.

## Introduction

Throughout the COVID-19 pandemic, unique characteristics of the novel virus continue to emerge daily. The typical symptoms have been described, which primarily encompass the upper respiratory manifestations. Extrapulmonary manifestations include gastrointestinal symptoms, such as anorexia, vomiting, and diarrhea as well as hepatic manifestations of abnormal aminotransferases [1]. However, severe rhabdomyolysis secondary to SARS-CoV-2 appears to be less prevalent, and thus, treatment of the disease is uncertain. Rhabdomyolysis is the breakdown of skeletal muscle tissue, a result of direct or indirect muscle injury. Pathophysiology is primarily caused by the injury to the muscle cell membrane or by depletion of energy; both of these pathways cause muscle necrosis and consequent release of intracellular components into the bloodstream [2]. The primarily measured serum markers include creatine kinase (CK), aldolase, and lactate dehydrogenase, which are all elevated [3]. Of the listed serum markers, the most specific is an elevation in the CK level greater than five times the upper limit of normal [2]. Physical etiologies of rhabdomyolysis include trauma, muscle hypoxia, exertion, and temperature changes. Non-physical causes can be attributed to drugs/toxins, infections, electrolytes, endocrine, autoimmune, and genetic disorders [4]. Signs and symptoms of the disease include myalgia, reddish-brown urine, fatigue, and extremity swelling [2]. The diagnosis is essential as treatment is supportive, with management focused on preserving renal function and preventing worsening disease.

This case report is a presentation of non-exertional rhabdomyolysis in an otherwise healthy young, African American male. The diagnosis of SARS-CoV-2 in this patient creates a unique diagnosis of rhabdomyolysis secondary to this particular viral etiology. The elevated CK level prompted extensive workup, with other possible causes excluded. This case offers perspective into the effects of the virus on systems other than the lungs, as commonly described in the literature. The treatment plan focuses on treating both the cause, the viral infection, and the severe complication.

## Case presentation

The patient is a 33-year-old African American male with no significant past medical history who presents to the Emergency Department for one day of hematuria. He also endorses a five-day history of myalgias, intractable headache, loss of taste, and minimal dyspnea on exertion. The patient was seen at local urgent care the night prior to presentation for the majority of similar symptoms and received testing for SARS-CoV-2 with the COVID-19 Pro-Genex test. Labs collected included complete blood count (CBC) and comprehensive metabolic panel (CMP). He was later called by the urgent care and was informed his liver enzymes were elevated. Throughout the evening, he noticed dark red color of his urine, which prompted his visit to the ED. He denies urinary frequency, urgency, and dysuria. He notes extreme myalgia, which preceded the hematuria, approximately five days prior to presentation. Myalgias had been gradually worsening. He admits to taking 400mg of ibuprofen the day prior with no symptomatic relief. There is no recorded fever at home, but he endorses chills. History is notable for moving into a new apartment the prior weekend; however, his myalgias began prior to this, and he denies significant exertional efforts. He works in a healthcare setting.

In the ED, the patient had a heart rate of 124 beats per minute, temperature of 98.3 F, oxygen saturation 98% SpO_2_ on room air, and respiratory rate of 20 breaths per minute. He weighs 90.4 kg. Physical exam findings reveal an ill-appearing African American male with dry lips and mucous membrane, lung sounds diminished at posterior bases, and the rest of the exam was unremarkable (Table 1).

**Table 1:** Pertinent Lab Results on Admission Abbreviation: ALT, alanine aminotransferase; AST, aspartate aminotransferase; CK, creatine kinase; hpf, high-power field; LDH, lactate dehydrogenase; RBC, red blood cells; WBC, white blood cells

Variable	Reference range	Value
Hematology		
Hematocrit (%)	36-46	47.3
Hemoglobin (g/dL)	12-16	15.6
RBC (×10^6^/µL) WBC (×10^3^/µL)	4.5-5.9 4.8-10.8	6.03 4.0
Platelets (×10^3^/µL)	130-400	143
Prothrombin time (s)	11.5-14.5	13.1
Prothrombin-time international normalized ratio	0.9-1.1	1.14
Blood chemistry		
Sodium (mmol/L)	135-145	134
Potassium (mmol/L)	3.4-4.8	3.2
Chloride (mmol/L)	100-111	104
Carbon dioxide (mmol/L)	23-28	28
Urea nitrogen (mg/dL)	8-25	11
Creatinine (mg/dL)	0.6-1.5	1.0
Glucose (mg/dL)	70-110	126
AST (U/L)	0-48	1,835
ALT (U/L)	13-60	190
Alkaline phosphatase	40-140	47
Bilirubin (mg/dL)	0.2-1.0	0.40
CK (U/L)	26.0-192.0	362,445
C-reactive protein (mg/dL)	0.00-0.60	4.32
Sedimentation rate (mm)	0-15	16
Adolase (U/L)	1.5-1.8	1224.3
Lactate dehydrogenase (U/L)	84.0-246.0	>4,000
Urine		
RBC, urine (per hpf)	<4	3-6
WBC, urine (per hpf)	<4	Negative
Urine blood	Negative	Large
Urine nitrate	Negative	Negative
Specific gravity	1.003-1.030	1.020
Protein	Negative	+3

Chest radiograph demonstrates a left lower lobe infiltrate. Computed tomography of the chest without contrast shows patchy infiltrates involving the bilateral lower lobe. COVID-19 SARS-CoV-2 assay was ordered inpatient. In the ED, further labs were drawn. Droperidol 2.5mg IV once was administered for his headache, however, acute dystonia developed and diphenhydramine 50mg IV was administered with a resolution of symptoms. The patient was admitted with the diagnosis of severe rhabdomyolysis, systemic inflammatory response syndrome, elevated liver function tests, hematuria, and suspected COVID-19 infection.

The patient was aggressively started on IVF with lactate ringer at 200mL per hour after CK level returned severely elevated (the lab was drawn prior to dystonia); strict ins and outs and renal function were monitored closely. Extensive lab testing was performed to rule out potential rhabdomyolysis causes. Epstein-Barr virus (EBV), HIV, cytomegalovirus IgM, toxoplasmosis, and acute hepatitis panel were negative for viral etiology. EBV IgG was positive. Acetaminophen level and urine drug screen were unremarkable. Rheumatological work up including antinuclear antibody (ANA), antineutrophil cytoplasmic antibody (ANCA), anti-Smith, SS-A/SS-B, anti-Jo 1, and gamma-glutamyl transferase (GGT) levels was negative. LDH > 4,000 U/L. CRP and sedimentation rate were elevated. Aldolase elevated 1,224.3 U/L. COVID-19 polymerase chain reaction (PCR) was positive within 24 hours of initial testing. Of note, urine myoglobin level is not reported in this report due to hospital-associated lab errors.

**Figure 1:**
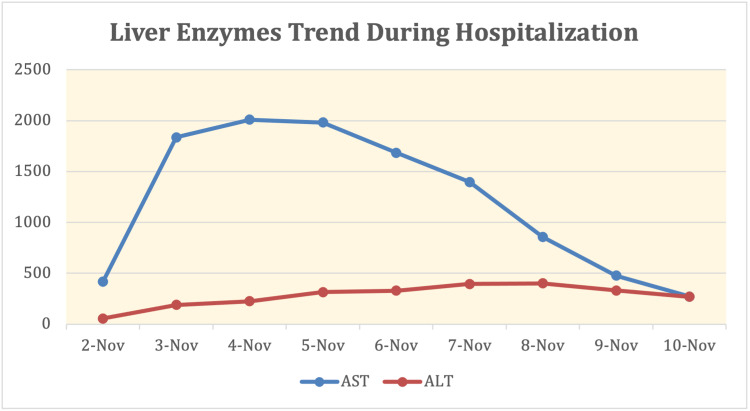
Liver Enzymes Trend During Hospitalization Abbreviation: AST, alanine aminotransferase; ALT, asparte aminotransferase

**Figure 2:**
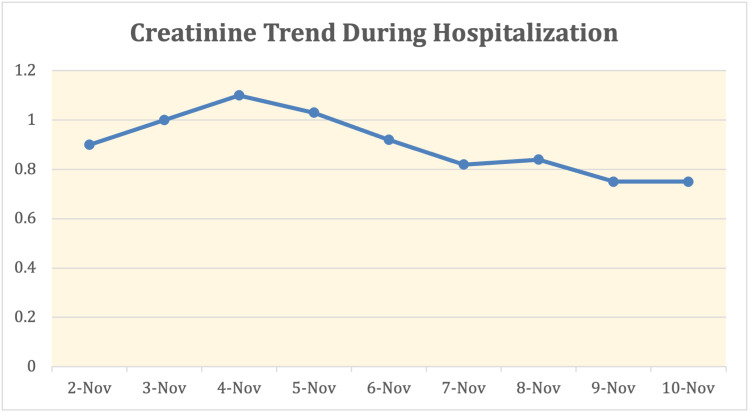
Creatinine Trend During Hospitalization

**Figure 3:**
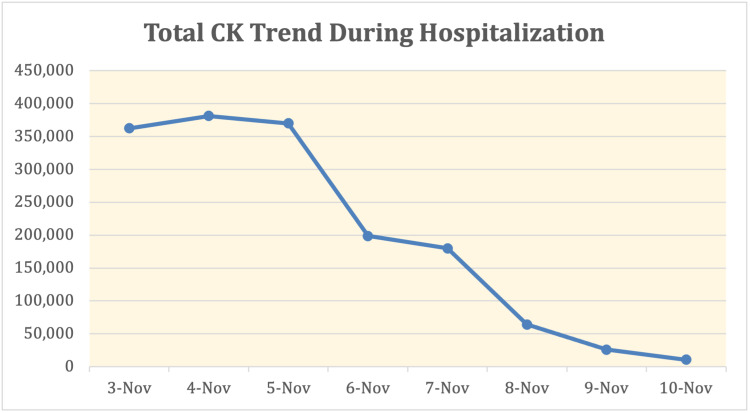
Total CK Trend During Hospitalization CK, creatine kinase

Nephrology and Infectious disease teams were consulted on the day of admission. Transaminitis was concluded to be secondary to rhabdomyolysis as no other acute liver etiologies could be identified. For suspected, early COVID-19 pneumonia (based on chest imaging), he was started on remdesivir 200mg IV on the first day, then remdesivir 100mg IV daily after for four days, for a total five-day course. Per COVID-19 pneumonia guidelines, he received vitamin C 500mg PO daily and zinc 500mg PO daily for five days, in addition to dexamethasone 6mg IV daily for 10 days. He was also started on a five-day course of azithromycin 500mg IV daily and Rocephin 2g IV daily for possible superimposed bacterial pneumonia with the left lower lobe infiltrate and developed symptoms of cough. Due to the elevated risk of thromboembolism with COVID-19, he received deep vein thrombosis (DVT) prophylaxis with enoxaparin 40mg subcutaneous daily during his hospitalization. CPK levels, liver enzymes, and renal function were trended daily (Figures 1-3).

On day 2 of the hospitalization, the patient was requiring fluid management included 1/2 normal saline + bicarbonate at 200 cc/hour. He was maintained on a bicarb drip for the rest of his hospitalization with improvement in his CK level and renal function. No further worsening of respiratory status during his stay and his dyspnea resolved prior to discharge. On day 7, he was discharged home with close follow-up with his primary care provider.

## Discussion

Workup excluded all other possible causes of rhabdomyolysis. The unique diagnosis of rhabdomyolysis in association with SARS-CoV-2 was confirmed in the setting of his positive SARS-CoV-2 assay inpatient test. The positive viral test demonstrated the association of the two conditions but did not confirm causation in this case, as rhabdomyolysis can be idiopathic. CK levels are markedly elevated, the peak value noted to be 389,081, however pulmonary manifestations are mild. Two notable cases involved with similarly elevated CK levels while infected with SARS-CoV-2 involved two African American males, one 19-year-old and one 16-year-old, with CK levels noted at 694,200 U/L and 426,700 U/L respectively [5,6]. In both cases, the respiratory status remains stable despite aggressive fluid administration. For our patient, the presentation was typical for rhabdomyolysis, confirmed with an elevation of CK greater than five times the upper limit of normal. The detection of myoglobin is also specific for rhabdomyolysis, but due to the short half-life of approximately two to three hours, it is only useful for detection in the early course of disease [7]. An extensive workup was performed to rule out other causes of rhabdomyolysis. Another distinctive finding, in this case, is the elevated liver enzymes associated with rhabdomyolysis. Acute hepatitis with a direct hepatocellular injury can be found in SARS-CoV-2 infection, as with other viral infections [3]. It is unclear whether elevated AST/ALT was secondary to rhabdomyolysis or COVID-19 infection in this case. Aldolase, GGT and a right upper quadrant ultrasound helped rule out potential obstructive pathology as a cause of aminotransferase elevation. Aldolase was elevated at 1,224.3 U/L while GGT and right upper quadrant ultrasound were negative, further supporting elevation in AST and ALT are less likely secondary to biliary obstruction or intrinsic pathology. This permitted use of remdesivir as treatment, despite this medication's side effect of further elevating AST/ALT. One study reports that in the setting of rhabdomyolysis, the abnormal rise in aminotransferases is typically not a direct sequela of primary hepatic injury [8]. In contrast, elevated AST/ALT occurs in 5%-50% of patients with COVID-19 virus, hypothesized to be a result of direct hepatocellular injury [9]. This is an important distinction to guide management accordingly. Obtaining a GGT value, which indicates liver injury and/or obstructive pathology, is another helpful marker to guide management [10]. The negative right upper quadrant ultrasound and negative GGT permitted the use of remdesivir in this case, as there was less concern for primary liver pathology. AST and ALT continued to be trended during the hospitalization and decreased at the same rate as the decrease in serum CK. One study has found that the elevation of AST/ALT over 1,000 U/L can be associated with increased mortality [11].

In the setting of severe rhabdomyolysis, IV hydration superseded the concern of inducing hypervolemia in the setting of the COVID-19 infection. Literature is controversial regarding the use of IV fluids in patients affected by the virus, due to the risk of acute respiratory distress syndrome (ARDS) with fluid resuscitation [12]. However, the consequences of rhabdomyolysis, such as hypovolemia leading to renal failure, electrolytes disturbance, and/or disseminated intravascular coagulation surpass the decision to hold fluid therapy. IV bicarbonate was used in response to an initial uptrend in creatinine. The goal with this fluid was to alkalize the urine to prevent further renal damage, as acidic urine can be associated with increased myoglobin renal toxicity [2]. Ultimately, we present this case to highlight the importance of aggressive fluid resuscitation to treat rhabdomyolysis, despite patient COVID-19 status. Importantly, the patient did not need further imaging of his chest after IV fluids were administered because respiratory status remained stable, on room air, throughout the course of the hospitalization. He was indeed started on antibiotic therapy and recommended treatment for COVID-19 pneumonia due to pulmonary infiltrates on imaging. The consequence of ARDS was not present, contrary to popular belief. Interestingly, the two other cases of severe rhabdomyolysis while infected with SARS-CoV-2 mentioned above showed similar sequelae with no respiratory complications despite aggressive IV fluids resuscitation, similar to this patient’s situation [5,6]. This case, as with the other two previously referenced, permits future investigation into whether severe rhabdomyolysis in younger patients with SAR-2-Coronavirus infection can be less likely linked to consequential pulmonary manifestations.

Certain risk factors are associated with increased mortality rate, including patients with hypertension, low levels of high-level data link control (HDLC), elevated white blood cells, age >60 years, hypertriglyceridemia, and hyperphosphatemia [13]. The recommended rate of crystalloid fluid initiation is at a rate of 1.5L per hour to maintain an adequate urine output of approximately 200-300 cc per hour [2]. Ultimately, the patient in this case did not decompensate from a respiratory standpoint, irrespective of the aggressive fluid resuscitation. He maintained his saturation on room air. CK levels continued to downtrend, and he was deemed stable for discharge with close follow-up.

## Conclusions

This case is important as it presents a rare case of rhabdomyolysis associated with SARS-CoV-2 in an otherwise healthy young adult, treated with liberal IV fluids. This presentation of rhabdomyolysis and superimposed transaminitis highlights the necessity of aggressive fluid resuscitation, despite the controversial idea that IV fluid resuscitation in COVD-19 patients is concerning for the development of ARDS. Therapy revolved around monitoring CK levels and early fluid administration to prevent the development of renal complications. The involvement of nephrology specialists early during the presentation helped guide fluid administration appropriately. Ultimately, identification of myalgias in SARS-CoV-2 positive patients as rhabdomyolysis can be lifesaving and lead to positive outcomes, if recognized early and treated appropriately. We hope further studies can shed light on the use of aggressive fluids in patients with rhabdomyolysis with concurrent SARS-CoV-2 infection, particularly those with mild respiratory disease and no sequelae of respiratory distress, despite aggressive IV fluids.
